# Dissemination of *Piscine orthoreovirus-1* (PRV-1) in Atlantic Salmon (*Salmo salar*) during the Early and Regenerating Phases of Infection

**DOI:** 10.3390/pathogens9020143

**Published:** 2020-02-20

**Authors:** Kannimuthu Dhamotharan, Håvard Bjørgen, Muhammad Salman Malik, Ingvild B. Nyman, Turhan Markussen, Maria K. Dahle, Erling Olaf Koppang, Øystein Wessel, Espen Rimstad

**Affiliations:** 1 Department of Food Safety and Infection Biology, Norwegian University of Life Sciences, 0454 Oslo, Norway; dhamubfsc@gmail.com (K.D.); muhammad.salman.malik@nmbu.no (M.S.M.); ingvild.nyman@nmbu.no (I.B.N.); turhan.markussen@nmbu.no (T.M.); oystein.wessel.finstad@nmbu.no (Ø.W.); 2 Department of Basic Science and Aquatic Medicine, Norwegian University of Life Sciences, 0454 Oslo, Norway; havard.bjorgen@nmbu.no (H.B.); erling.o.koppang@nmbu.no (E.O.K.); 3 Department of Fish Health, Norwegian Veterinary Institute, 0454 Oslo, Norway; maria.dahle@vetinst.no

**Keywords:** PRV-1, *Piscine orthoreovirus*, HSMI, pathogenesis

## Abstract

*Piscine orthoreovirus-1* (PRV-1) can cause heart and skeletal muscle inflammation (HSMI) in farmed Atlantic salmon (*Salmo salar*), but the line of events from infection, pathologic change, and regeneration has not been thoroughly described. In this study, the cellular localization and variation of PRV-1 RNA and protein levels were analyzed at different times post-exposure in experimentally infected Atlantic salmon. Immunohistochemistry, flow cytometry, and Western blot were used for assessment of the presence of the PRV-1 σ1 protein, while RT-qPCR and in situ hybridization were performed for viral RNA. Histopathologic evaluation demonstrated that PRV-1 infection induced heart lesions typical of HSMI, such as severe epicarditis and myocarditis with degeneration of cardiomyocytes, necrosis, and diffuse cellular infiltration. PRV-1 infection of erythrocytes and the peak viral plasma level preceded virus presence in cardiomyocytes and hepatocytes. Arginase-2-positive, macrophage-like cells observed *in* the heart indicated possible polarization to M2 macrophages and the onset of regenerative processes, which may contribute to the recovery from HSMI. The virus was cleared from regenerating heart tissue and from hepatocytes, but persisted in erythrocytes.

## 1. Introduction

Heart and skeletal muscle inflammation (HSMI) is an important viral disease in farmed Atlantic salmon (*Salmo salar*), first reported in Norway in 1999. HSMI occurs mainly in the marine phase, but incidences have also been reported from broodstock farms and hatcheries [1]. HSMI is caused by *Piscine orthoreovirus* (PRV-1) [2]. The virus, recognized as a species in the genus *Orthoreovirus*, family *Reoviridae*, packs its 10-segmented dsRNA genome in double-layered, spherically-shaped icosahedral particles with 80 nm diameters [3]. PRV-1 is ubiquitous in the marine phase of farmed Atlantic salmon, but is also prevalent in wild Atlantic salmon [4]. PRV was first discovered using next-generation sequencing in 2010 [5]. The virus has resisted cultivation in cell lines, which has been challenging for the study of the infection dynamics. However, virus can be purified from infected blood cells by gradient ultracentrifugation, and has been used in challenge models demonstrating that PRV-1 is the cause of HSMI in Atlantic salmon [2]. Moreover, PRV is also widespread among farmed and wild Pacific salmonids, such as coho (*Oncorhynchus kisutch*) and Chinook salmon (*O. tshawytscha*) [6,7]. There are three known subtypes of PRV, of which PRV-1 causes disease in Atlantic salmon [2], PRV-2 in coho salmon [8], and PRV-3 in rainbow trout [9].

In Atlantic salmon pens, fish suffering from HSMI are anorexic and show aberrant swimming patterns, and the accumulated mortality in disease outbreaks range from 0–20%. However, the morbidity of histopathological changes may be up to 100% [10]. Challenge studies found that infected fish develop histopathological changes in the heart, but no mortality [2]. Histological investigations show focal or diffused lesions, with infiltration of inflammatory cells in the epicardium, myocardium, and vacuolation, as well as loss of striation, necrosis, and the infiltration of mononuclear cells in red skeletal muscle. Multifocal necrosis in liver, focal hemorrhage, and the accumulation of erythrocytes in the spleen and kidney are commonly observed in severely infected fish [11]. 

Erythrocytes are the main target cells for PRV, and circulating infected erythrocytes are found in any organ [12]; nevertheless, PRV-1 in Atlantic salmon does not cause anemia [3]. In experimental infections, there is a transient peak in PRV-1 protein level in erythrocytes 3–5 weeks post-infection, followed by a sharp drop, while the viral RNA level stays relatively high [13]. dsRNA is the dominant form of RNA in the blood cells, particularly during the persistent stage of infection [14,15].

In Chinook salmon, PRV-1 is associated with jaundice syndrome and anaemia, but causation studies are required for further confirmation. Affected fish develop degenerative necrosis in the liver and kidneys, and necrotic hepatocytes contain high levels of hemoglobin [16]. Erythrocytic inclusion body syndrome (EIBS) in coho salmon in Japan is caused by PRV-2 [8]. The infected fish have viral inclusions in 80%–100% of erythrocytes, and the hematocrit value is significantly reduced, causing anemia-associated mortalities [17]. PRV-3 infects erythrocytes and is shown to cause heart pathology in Rainbow trout [9,18]; it is also associated with jaundice in coho salmon [19]. Anemia is observed in PRV-3-infected Rainbow trout [20], but not consistently [21].

Within PRV-1, there two major strains that can be differentiated based on the S1 and M2 segments. One strain is associated with HSMI, and is currently dominating in Norwegian salmon aquaculture, while the second strain is less associated with HSMI and is present in the North American Pacific and historical samples from Norway [22]. In the present study, we have used a current Norwegian PRV-1 strain associated with HSMI. The various diseases and histopathological changes caused or associated with the subtypes of PRV are mainly linked to clinical signs arising from effects on erythrocytes (anemia, EIBS), the heart (HSMI), and the liver (jaundice). Hence, the characterization of infected cells, amount of viral RNA, and protein levels and mechanisms of infection of the heart and liver could give more information on viral localization and temporal changes of the infection. In this study, we tracked and visualized PRV-1 RNA and proteins in Atlantic salmon erythrocytes, hearts, and livers, as well as arginase-2 in the heart. The latter is an important marker in fish of M2 macrophages, which play a role in resolving inflammation [23]. Immunohistochemistry, flow cytometry, and Western blotting were used to asses PRV-1 protein levels, and RT-qPCR and in situ hybridization were used to target viral RNA. This was combined with histologic evaluation.

## 2. Results

### 2.1. PRV-1 in Blood Cells

**Figure 1:**
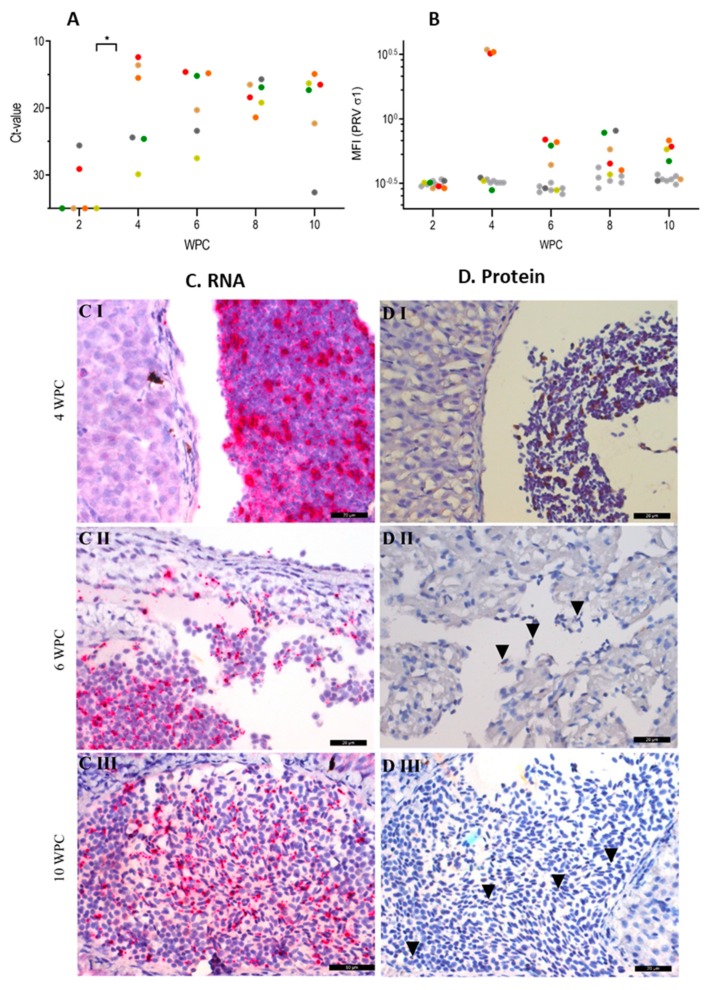
*Piscine orthoreovirus* (PRV-1) RNA and protein in blood cells. (**A**) RT-qPCR. (**B**) Flow cytometry. (**C**) In situ hybridization. (**D**) Immunohistochemistry.

In blood cells, PRV-1 RNA was first detected at 2 weeks post challenge (wpc) by qPCR in two out of six fish, at relatively low levels (cycle treshold (Ct) 25.6 and 29.1) (Figure 1A). At 4 wpc, the viral RNA load had increased to a mean Ct value of 20.0 ± 7.1 in the six fish, of which three had high loads of PRV-1 RNA (Ct 12.4 to 15.5). Thereafter, the viral RNA loads remained consistently high until the end of the study at 10 wpc (Ct value = 19.9 ± 6.6).

PRV-1 RNA detected by RT-qPCR is shown as mean Ct values for individual fish represented by coloured dots in Figure 1A. Brackets and asterisks denote statistical difference between 2 wpc and 4 wpc. In Figure 1B, PRV-1 σ1-protein detection by flow cytometry is shown as the mean fluorescence intensity (MFI) for individual fish (dots) at each time point. The PRV-1 infected- and control groups are indicated by colored and grey colors, respectively. In Figure 1C, PRV-1 RNA detected in blood cells by in situ hybridization (red color) at 4 wpc (I), 6 wpc (II), and 10 wpc (III) is shown. Lastly, Figure 1D shows PRV-1 σ1-protein detected by immunohistochemistry (brown color), with arrowheads to indicate positive cells at 4 wpc (I), 6 wpc (II), and 10 wpc (III). Peripheral blood aggregates are observed in the liver (CI, DI, CIII, DIII) and in the heart (CII, DII). Representative sections are presented. Scale bars are 20 µm.

PRV-1 RNA in blood cells was also detected through in situ hybridization (ISH) of tissue sections (Figure 1C). In general, the staining of PRV-1 in blood cells following ISH was in accordance with the kinetics observed by qPCR. At 4 wpc, many peripheral blood cells in the heart and liver, assessed mainly as erythrocytes due to their morphology, were heavily stained for PRV-1 RNA (Figure 1(CI)). The PRV-1 positive blood cells were observed as peripheral cells within the cardiac chamber. At 6 wpc, both peripheral and infiltrating blood cells in the heart myocardium were PRV-1-positive (Figure 1(CII)). Blood cells continued to stain heavily for PRV-1 by ISH at 8 and 10 wpc (Figure 1(CIII)), correlating with the low qPCR Ct-values (15.7–23.7) in blood.

Like PRV-1 RNA, high loads of the PRV-1 σ1 protein were observed by flow cytometry in three out of six fish at 4 wpc (Figure 1B). This was consistent with the detection of PRV-1 by immunohistochemistry, where positive blood cells were found in blood clots in the heart (not shown) and liver (Figure 1(DI)). However, post-4 wpc, the PRV-1 protein level dropped substantially, as observed by flow cytometry (Figure 1B). Furthermore, only a limited number of weakly stained blood cells could be observed by immunostaining 6–10 wpc (Figure 1(DII,DIII)). This contrasts the persistent high levels of PRV-1 RNA in this period.

**Figure 2:**
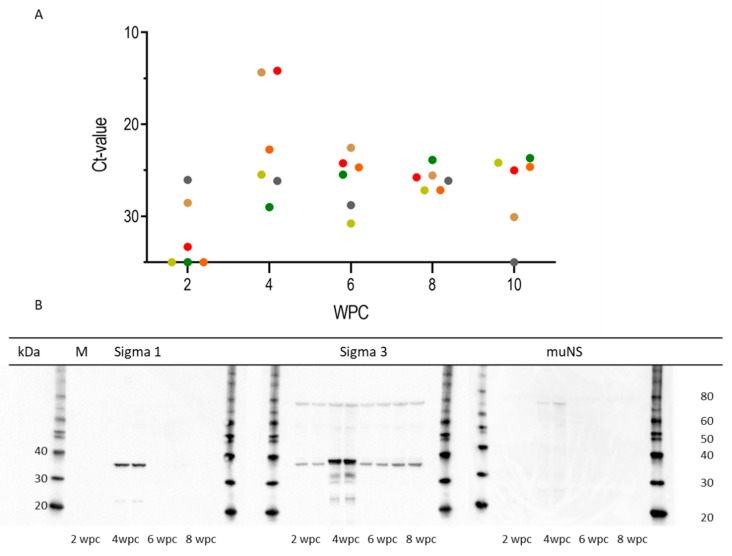
PRV-1 in plasma.

The viral RNA load in plasma measured by RT-qPCR at 2, 4, 6, 8, and 10 wpc is shown in Figure 2A, presented as mean individual Ct values at each time point (*n* = 6) for individual fish; fish are represented by coloured dots. Figure 2B shows viral protein loads in plasma detected by Western blotting, using antibodies targeting the PRV-1 σ1, σ3, and µNS proteins. Samples from two fish taken at 2, 4, 6, and 8 wpc were analysed. PRV-1 proteins were detected in plasma from 4 wpc only. In the σ3 blot, a band with a slightly lower molecular weight than σ3 was observed with all the samples, considered to be background staining, as a similar background has been observed previously with this antiserum [14]. At 4 wpc, the σ3 stands out, and breakdown products can also be seen. Stronger staining was observed for σ1 and σ3 compared to that of µNS. The specificities of the antibodies in Western blot have been demontrated for the PRV-1 σ1 and σ3 proteins in [14], as well as for the µNS protein in [13].

### 2.2. PRV-1 Load in Plasma 

The viral RNA load in plasma peaked at 4 wpc, with a mean Ct value of 21.9 ± 6.3, coinciding with the peak of viral protein in blood cells. There was substantial variation between individual fish. Two fish had especially low Ct values of 14.3 and 14.1, with correspondingly low Ct values for blood cells (Figure 1A and Figure 2A). Although the viral RNA load in plasma decreased between 4 and 6 wpc, it was rather stable thereafter, and was not cleared by 10 wpc (mean Ct value of 27.0 ± 4.5)—i.e., at the end of the experiment. PRV-1 proteins in plasma, tested by using antibodies against σ1, σ3, and µNS proteins, were detected by Western blotting only at 4 wpc (Figure 2B). 

### 2.3. PRV-1 Load in the Heart

**Figure 3:**
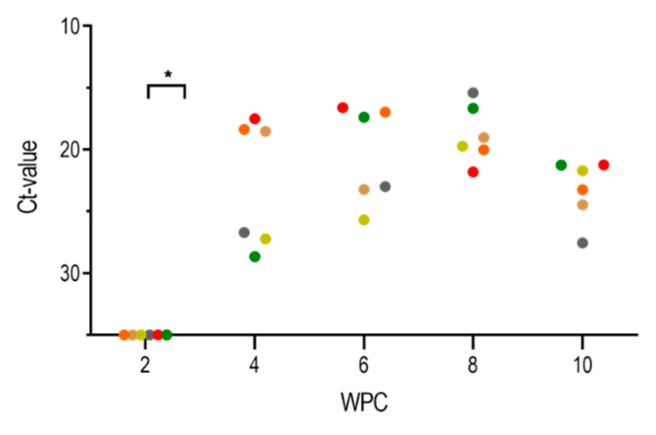
PRV-1 RNA in the heart.

**Figure 4:**
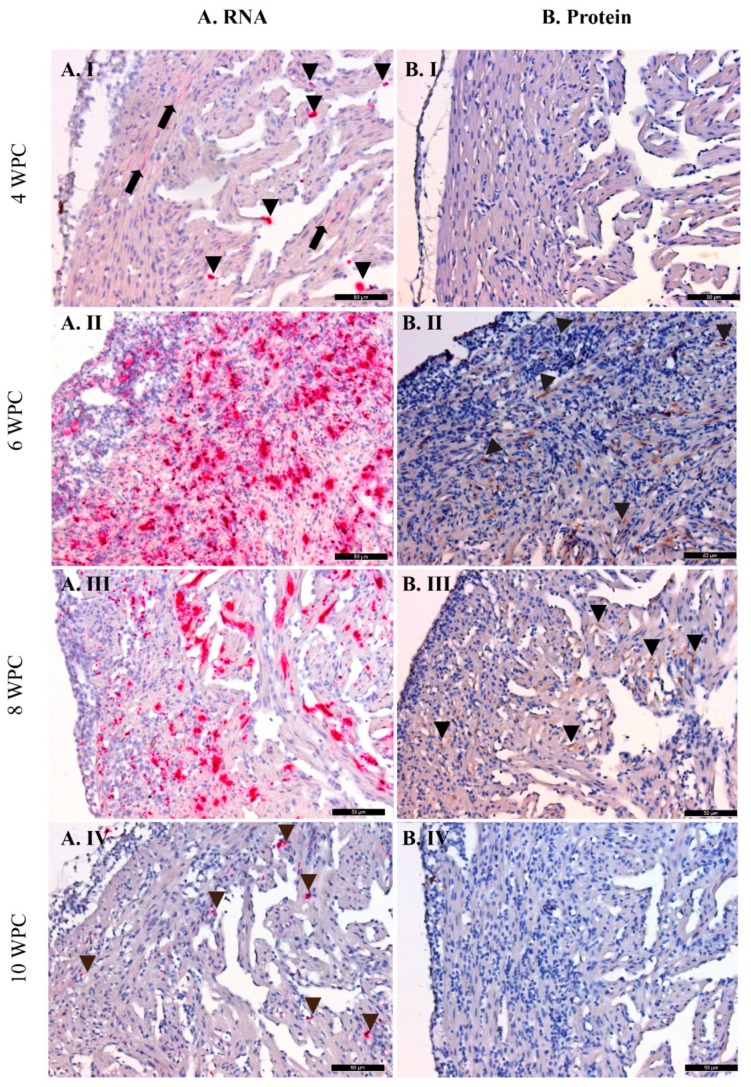
PRV-1 RNA and protein in the heart.

In the heart, PRV-1 RNA was first detected by RT-qPCR at 4 wpc (mean Ct value of 22.8 ± 5.2) (Figure 3). Similarly, PRV-1 RNA was detected by ISH in the heart at 4 wpc, demonstrated by less intense and punctate staining of myocardial cells in the stratum compactum and spongiosum (Figure 4(A.I)). In addition, a number of PRV-1 positive blood cells were observed apparently attached to the endothelial lining of the endocardium. At 6–8 wpc, peak staining by ISH was observed in cardiomyocytes, including the ventricle epicardium and in myocardial cells in the compactum and spongiosum (Figure 4(A.II,A.III)). The viral RNA load decreased thereafter, with a mean Ct value of 23.2 ± 2.4 at 10 wpc (Figure 3) with only a few cardiomyocytes positively stained by ISH. However, PRV-1 was observed by ISH in intravasal erythrocytes and in infiltrating leukocyte-like cells at 10 wpc (Figure 4(IV)). Positive and negative controls for ISH are shown in Supplementary Figure S1.

PRV-1 RNA in the heart was detected by RT-qPCR, shown in Figure 3 as mean Ct values of individual fish (dots) at each time point. Brackets and asterisks denote statistical differences between 2 wpc and 4 wpc.

Figure 4A shows ISH staining of PRV-1 RNA, with Figure 4(A.I) showing peripheral blood cells in heart tissue (PRV-1 positive cells marked by arrowheads). Compact myocardial cell PRV-1 staining (arrow) appears as cytoplasmic dots. In Figure 4(A.II,A.III), heavy infection and staining is seen in ventricle epcicardium and endocardial cells, along with severe epicarditis and myocarditis. Figure 4(A.IV) shows cardiomyocytes with less staining at 10 wpc in regenerating heart tissue with PRV-1-positive, infiltrating inflammatory cells (arrowheads) in spongy myocardium. Figure 4B displays IHC staining of PRV-1 σ1, with no staining seen at 4 wpc (Figure 4(B.I)); epicardium, compact, and spongy myocardium staining at 6 wpc (Figure 4(B.II)); immunostained cells in spongy myocardium at 8 wpc (Figure 4(B.III)); and no positive staining in cardiomyocytes at 10 wpc (Figure 4(B.IV)). PRV-1-positive cells are marked by arrowheads. The scale bars are 50 µm.

In contrast to detection of PRV-1 RNA, PRV-1 σ1 protein was present for a much shorter window of time, as demonstrated by IHC. No viral proteins could be detected in the cardiac tissue at 2–4 wpc (Figure 4(B.I)). At 6 wpc, PRV-1 protein was detected in cardiomyocytes in the ventricle, where both compact and spongious compartments of the myocardium were strongly stained by IHC (Figure 4(B.II)). At 8 wpc, the staining was reduced both by the number of positive cells and in intensity (Figure 4(B.III)). By 10 wpc, no positive cardiomyocytes could be detected (Figure 4(BIV)). Bulbous arteriosus was negative for staining at all sampling points. Positive and negative controls for IHC are shown in Supplementary Figure S2.

### 2.4. Histological Findings

**Figure 5:**
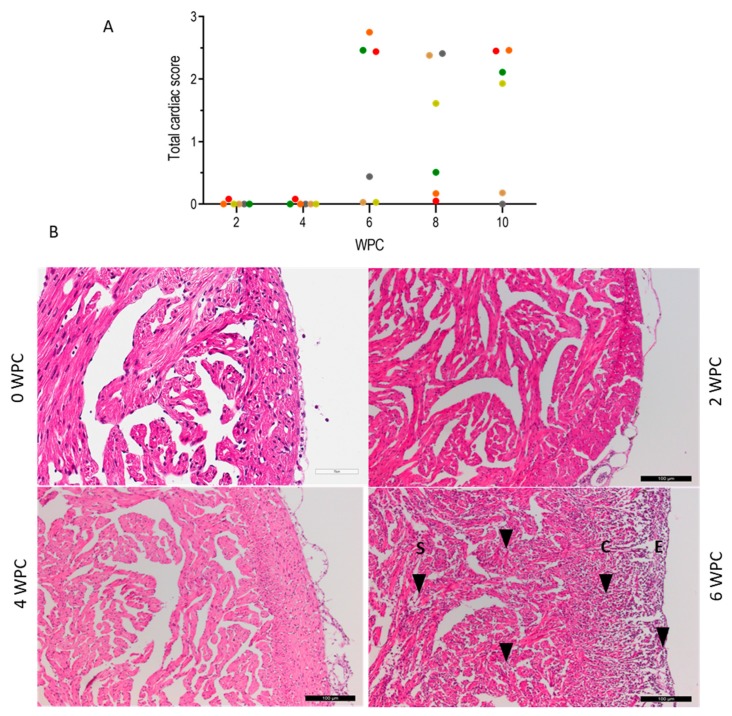
Heart histopathology.

No histopathological changes were observed in the heart or liver at 2 and 4 wpc (Figure 5A). At 6 wpc, severe epicarditis and myocarditis was observed (Figure 5A). This included the degeneration of cardiomyocytes, loss of tissue architecture, necrosis, and diffused cellular infiltration in the epicardium and myocardium (Figure 5B). At 6 wpc, three out of six fish had a cardiac pathology score higher than 2 (scale: 0–3) (Figure 5A), corresponding in time to low Ct values in the heart. Most of the liver sections had normal histoarchitecture; however, some of the hepatocytes showed minor degenerative changes in the late stage of infection, i.e., after 6 wpc. 

The histopathological heart score is shown for individual fish (dots) at 2, 4, 6, 8, and 10 wpc (*n* = 6) in Figure 5A. Heart tissue from Atlantic salmon infected with purified PRV-1 is shown in Figure 5B. There were no changes observed at weeks 2 and 4, while from 6 wpc histopathological changes in accordance with HSMI appeared. These were severe degeneration, infiltration of inflammatory cells (highlighted by arrowheads in Figure 5B), vacuolization, necrosis in the epicardium (E), compact (C), and spongy (S) myocardium, and loss of tissue architecture, which are visible in hematoxylin and eosin (H&E) stained tissues. The scale bars are 100 µm.

### 2.5. In Situ Hybridization of Arginase-2 in the Heart

**Figure 6:**
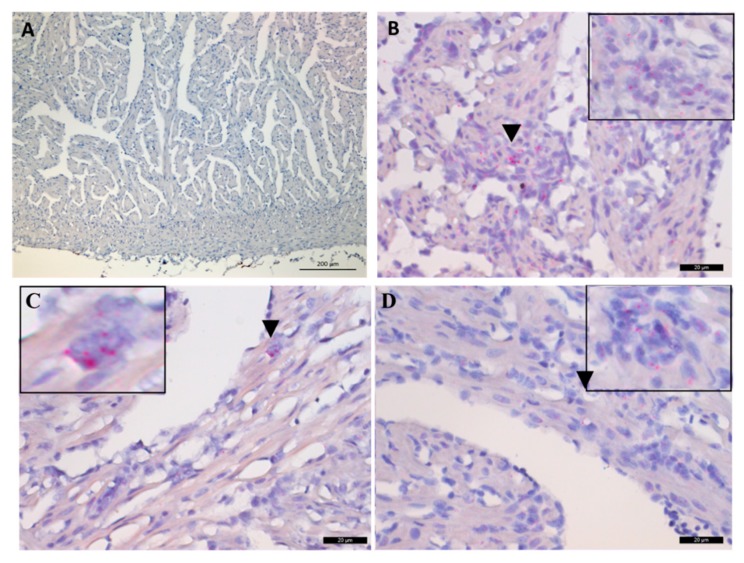
In situ hybridization (ISH) staining of the Atlantic salmon arginase-2 (arg-2) transcripts in heart tissues. (**A**) 4 wpc, (**B**) 6 wpc, (**C**) 8 wpc, (**D**) 10 wpc.

At 2 and 4 wpc, no ISH staining for the regenerating (M2) macrophage marker arginase-2 (arg-2) was observed in the heart tissue (Figure 6A). Positively stained infiltrating macrophage- or leukocyte-like cells were observed from 6 wpc at the peak of the histopathological changes (Figure 6B–D). The highest number of arg-2 positive cells were observed at 6 wpc (Figure 6B). 

A. No arginase-2 RNA staining was observed at this time point, 4 wpc. Scale bar is 200 µm. B–D. ISH staining of arginase-2 RNA at 6, 8 and 10 wpc as indicated. Some of the infiltrating cells are stained (arrowheads). A selected region is shown at magnification (top left/right corner). Scale bars are 20 µm.

### 2.6. PRV-1 Load in the Liver

**Figure 7:**
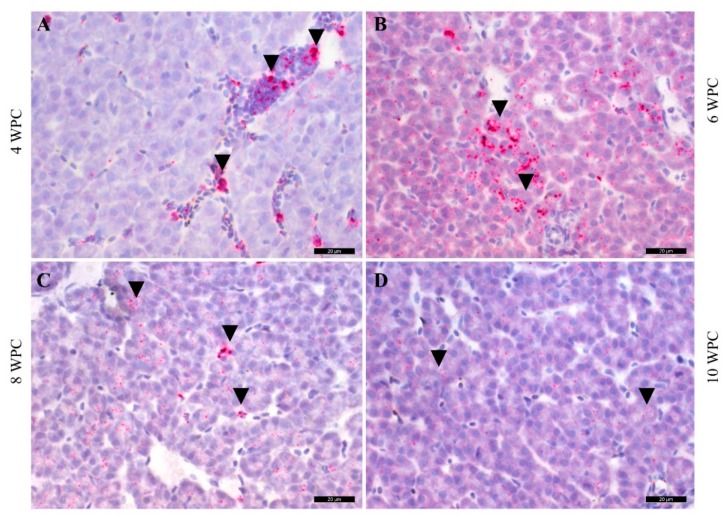
ISH staining of PRV-1 RNA in liver. (**A**) 4 wpc, (**B**) 6 wpc, (**C**) 8 wpc, (**D**) 10 wpc.

In the liver, no staining of PRV-1 RNA by ISH was observed at 2 wpc, whereas a few hepatocytes were positive at 4 wpc (Figure 7A). Intense focal staining of polygonal hepatocytes was observed at 6 wpc (Figure 7B), and staining was observed from 8 to 10 wpc at low intensity (Figure 7C,D). In the liver sections, hepatocytes were negative by IHC throughout the trial period (not shown).

As shown in Figure 7, eripheral blood erythrocytes (arrowheads) and neighboring hepatocytes were stained 4 to 10 wpc. The scale bars are 20 µm. 

## 3. Discussion

The aim of the present study was to monitor tissue distribution of PRV-1 in key target cells and organs during the early and regenerating phases of HSMI in Atlantic salmon. Blood, heart, and liver tissues were analyzed, due to their central role in PRV-1 pathogenesis in salmonids. All applied methods uniformly confirmed that erythrocytes are the main initial target cells for PRV-1. The viral replication in erythrocytes and peak plasmic viremia preceded infection of cardiomyocytes and hepatocytes. The erythrocytes were the first cells observed to be infected by PRV-1, and two out six fish were positive at 2 wpc, with no other cell types virus-positive at this stage. The PRV-1 load in erythrocytes peaked at 4 wpc, as observed by flow cytometry, IHC, and ISH. At 4 wpc, viral RNA and viral protein levels also peaked in plasma, indicating that the erythrocytes are an important source of virus in plasma. Both the structural proteins σ1 and σ3, as well as the nonstructural µNS protein were detectable in plasma at the peak of infection—in this case, at 4 wpc. The µNS protein, thought to play a major role in viral factory structure [24], was only present in infected cells, and was not a part of virus particles. It was found in lower amounts in plasma compared to the structural proteins. The polyclonal antiserum that was used against µNS has previously been shown to display similar sensitivity as the sera used against σ1 and σ3 [13]. The presence of nonstructural viral proteins in plasma at the peak of PRV-1 infection in Atlantic salmon suggests that some degree of erythrocyte lysis has occurred. This is also in line with the detection of viral RNA transcripts at the peak of infection [14]. Anemia is not a common clinical sign for PRV-1 in Atlantic salmon, indicating that compensatory mechanisms handle the hemolysis, as opposed to the anemia caused by PRV-2 infection in coho salmon [8]. In the heart and liver, ISH demonstrated positive staining of cells at 4 wpc, but the staining was mainly on the luminal side of vessels, indicating invasion of the virus to the tissue from the luminal side. 

The intraperitoneal injection of purified virus ensured an even infection dose in the experimental fish. Nevertheless, the differences in infection load in the blood between individual fish were large—i.e., at 4 wpc the Ct values in blood ranged from 12 to 30, and a high viral RNA load in cardiomyocytes could be seen by ISH at both 6 and 8 wpc. In a previous PRV-1 study, where a high virus dose was used for injection, the heart changes peaked already at 4 wpc, indicating that the virus exposure dose influenced the timing of the infection. Together, this demonstrates the contribution of both the infectious dose of virus and the host factors regarding timing and the severity of heart pathologic changes. Both the injection and mucosal routes of infection induce HSMI, indicating that the route of exposure is not essential for disease development [2]. However, injection does not reflect the natural port of entry. In the farming environment, virus uptake most likely occurs through mucosal surfaces. “REO” is an acronym for respiratory enteric orphan, and as the name implies, the gills and enteric system are the likely ports of entry. Anal administration has demonstrated intestinal uptake of PRV [25]. It is noteworthy that it took a relatively long time, i.e., 2 weeks after intramuscular injection of virus in the present study, before PRV-1 was detected in erythrocytes, which does not suggest an immediate infection of erythrocytes after exposure. In the marine farming situation, the infection kinetics will not be as uniform as in experimental set-ups. In net pen populations of up to 200,000 individuals per net, and with several nets at a site, the infection of the individual fish will not be synchronized, indicating virus shedding and spread for an extended period.

In our experimental trial, the viral protein load, assessed by the presence of the σ1 protein, declined rapidly in erythrocytes after 4 wpc, while viral RNA levels remained high. The results from all detection methods used, i.e., flow cytometry, IHC, ISH, and RT-qPCR, mutually supported this finding. The sharp decline in viral protein levels in erythrocytes has been observed also in previous studies where the viral proteins σ1, σ3, µ1, µNS, and λ1 were tested [13], and all followed the same pattern as σ1, which was used as a proxy for viral proteins in our study. After the peak of infection, the erythrocytic viral RNA level does not correspond with the level of viral protein [24], and in this study, similar observations were made for the heart and liver after 6 wpc. 

Salmon erythrocytes have a life span that is influenced by water temperature, fish activity, and many other factors [26]. The PRV-1 infection of erythroid progenitor cells has been indicated in recent studies [14]. Young erythrocytes have a more active translational machinery than old ones [27], and are probably better suited to be host cells for viral replication. In a previous study, viral RNA could be detected by RTqPCR in blood samples even 59 weeks after initial infection [28]. The low viral protein level and relatively high level of viral RNA, mainly in the form of dsRNA and not as transcripts during later stages of infection in erythrocytes [15], could suggest inhibition of both viral transcription and translation. Antiviral responses have reported that the PRV-1 infection of cultured Atlantic salmon red blood cells induces Mx and Protein kinase R (PKR) [29], the latter being a mediator of translational inhibition [30]. In mammalian orthoreovirus (MRV) infected cells, translational shutoff has been demonstrated through PKR and phosphorylation of the eukaryotic translation initiation factor (eIF)-2α, as part of the interferon-regulated antiviral response [31,32]. Further research would provide more insights into the molecular control mechanisms of PRV replication in target cells, and for the persistence of PRV-1 RNA in salmon erythrocytes, a well-suited cell type for long-term viral persistence. 

In the present study, Atlantic salmon injected with purified PRV-1 developed epicardial and myocardial changes typical of HSMI from 6 wpc. The cardiomyocytic changes could be attributed to the viral replication in these cells, or be secondary effects of the antiviral immune response. Furthermore, tissue damage would release cytokines or damage-associated molecules that could attract additional immune cells to the site. The peak viral load in the heart coincided with the infiltration of inflammatory cells and a high cardiac score, previously characterized as being dominated by CD8 positive T-cells [33]. This indicates that cytotoxic CD8 positive cells help to clear the virus from heart tissue. A previous study indicates that the PRV-1-specific antibody response also corresponds in time to clearance of the virus and heart regeneration [34]. The histopathological changes in the absence of detectable viral RNA in cardiomyocytes indicates efficient clearance of virus at 10 wpc. Despite the viral clearance in cardiomyocytes, the relatively low PRV-1 Ct values observed by RT-qPCR in heart tissue could be contributed to the presence of numerous infected circulating erythrocytes in heart vessels. It is important to note here that histopathological scoring of heart tissues is done independent of viral presence. During a field HSMI outbreak, severely affected fish with severe heart damage and associated circulatory failure succumb to death. However, most of the fish with severe heart histopathology and high viral loads will survive the infection, as indicated by the relatively low accumulated mortality commonly observed for field outbreaks of HSMI. This indicates that most fish recover through viral clearance and tissue regeneration, as teleost cardiomyocytes can regenerate after injury or infection [35]. It should be noted, however, that stress and handling could increase mortality from PRV-1 infection and HSMI [36].

Cytokines released from infected tissue can polarize and activate macrophages to participate in the regenerative processes [37]. Arginase-2 is considered a cell marker for regenerating macrophages in fish [23]. Here, arginase-2 expression in macrophage-like infiltrating cells was already observed in the heart from 6 wpc. Arginase converts L-arginine into L-ornithine and urea, and ornithine is the precursor for polyamines and prolines, which are assumed necessary for wound healing and regeneration [38]. The finding of abundant cells expressing arginase-2 in the heart already from 6 wpc suggests an early onset of regenerative processes, which may contribute to the relatively fast onset of recovery after HSMI. This indicates that severe HSMI outcomes in a field situation may be linked to an impaired ability to regulate the regeneration process.

In the present work, we observed that hepatocytes stained positive for PRV-1 RNA, and like the findings in the heart, the staining was mainly found at the luminal side of vessels in the liver during early infection. At 6 wpc, we found that hepatocytes were generalally infected at a low degree, as observed by weak but punctuate staining patterns, whereas a few hepatocytes were more heavily infected. Whether the weak staining in the hepatocytes mirrored the actual replicating virus in these cells or merely the engulfment of infected erythrocytes or erythrocytic debris is not clear. The ISH staining of liver remained low, and did not indicate heavy virus replication. We conclude that Atlantic salmon hepatocytes are susceptible, as some were heavily infected but not very permissive to the PRV-1 infection. The lack of subsequent infiltration of inflammatory cells in the liver, in contrast to observations in the heart, could indicate the differences in permissiveness between Atlantic salmon hepatocytes and cardiomyocytes for PRV-1 replication.

The lack of cell culture for isolation methods of PRV makes the findings in the present study helpful for the design of PRV challenge studies. Estimation of the load of infectious virus particles in the blood is difficult, due to differences in viral RNA and protein kinetics in blood cells. Weekly sampling and testing for virus proteins, with methods like flow cytometry, combined with molecular techniques would ease the estimation of the peaks of infection and the HSMI pathological lesions two weeks thereafter. The outcome of a PRV infection depends on the PRV subtype and the host salmonid species, as well as environmental and management factors [2,8,20]. In the present study, we found that PRV-1 replication in erythrocytes and peak plasmic viremia in Atlantic salmon precede infection of cardiomyocytes and hepatocytes. PRV-1 was cleared from cardiomyocytes and hepatocytes but persisted in erythrocytes. The regeneration of the heart from HSMI was associated with arginase-2-positive, macrophage-like cells. This makes an important contribution to the understanding of HSMI development and recovery.

## 4. Materials and Methods

### 4.1. Challenge Experiment

A challenge experiment was conducted at VESO Vikan aquatic research facility (Vikan, Norway). The experiment had been approved by the Norwegian Food Safety Authority (NFDA), according to the European Union Directive 2010/63/EU for animal experiments (permit number 11251). The experimental fish included 104 seawater-adapted Atlantic salmon (StofnFiskur Optimal strain) with an initial average weight of 100 grams. The fish were acclimatized for one week prior to challenge, fed according to standard procedures, and kept under a 24 h light regime. The fish population had been screened for PRV, infectious salmon anemia virus (ISAV), salmonid alphavirus (SAV), and infectious pancreatic necrosis virus (IPNV) before initiation of the study. They were kept in tanks supplied with particle-filtered and UV-treated seawater (34‰ salinity, 12 °C), and observed once a day at minimum. Prior to handling or sampling, fish were anesthetized by bath immersion in benzocaine chloride (2–5 min, 0.5 g/10 L water) or euthanized using concentrated benzocaine chloride (1 g/5 L water).

Eight fish were sampled prior to challenge, and then the remaining fish were divided into two groups. One of the group (48 fish) was challenged by 0.1 mL/fish intramuscular injection with 60,000 copies of the PRV-1 NOR 2012 strain, originating from an HSMI outbreak in Norway [2]. The virus had been purified according to previously published procedures [2]. The other group (48 fish) was injected with 0.1 mL of PBS (Thermo Fisher Scientific, Waltham, MA, USA). Eight fish from each group was sampled every two weeks post-challenge. Heparinized blood was collected from the caudal vein for PRV-1 analysis by RT-qPCR and flow cytometry. In addition, parallel tissue samples from heart and liver were sampled in RNAlater for RT-qPCR (Life Technologies, Carlsbad, CA, USA) and in 10% phosphate buffered formalin for histology, immunohistochemistry, and in situ staining.

### 4.2. RNA Isolation and RT-qPCR

From six fish, approximately 25 mg of heart tissue and 20 µL blood pellets were separately added to 650 µL QIAzol lysis reagent (Qiagen, Hilden, Germany) with 5 mm steel beads, and homogenized using TissueLyser II (Qiagen, Venlo, Netherlands) for 2 × 5 min at 25 Hz. After addition of 130 µL chloroform and centrifugation at 12,000 *g* for 15 min, the aqueous phase was transferred to QIAcube (Qiagen) for RNA extraction. Following the manufacturer’s instructions, the total RNA was eluted in 50 μL RNase-free water and the concentration was measured using a NanoDrop ND-1000 spectrophotometer (ThermoFisher Scientific, Waltham, MA, USA).

RNA was isolated from 10 µL plasma samples, which were diluted to 130 µL in phosphate buffered saline (PBS) prior to using a QIAmp viral RNA mini kit (Qiagen), following manufacturer’s instructions. The RNA was eluted in 50 µL elution buffer and stored at −80 °C. 

AQiagen OneStep RT-PCR kit (Qiagen) was used to perform RT-qPCR with 100 ng RNA from tissues or 5 µL RNA elution from plasma. The RNA was denatured at 95 °C for 5 min and then rapidly cooled on ice prior to RT-qPCR. Reverse transcription was conducted at 50 °C for 30 min, and PCR at 95 °C for 15 min, with 40 cycles of 94 °C/30 s, 55 °C/30 s, and 72 °C/30 s. The samples were run in duplicate, and a sample was defined as positive if both parallel samples had a Ct < 35, as used in previous studies [2,29,39]. The RT-qPCR targeted segment S1, which encodes the σ3 and p13 proteins.The primers and probes used in the PRV-1 specific assay have previously been described [3]. The statistical differences in the viral load were determined using one-way ANOVA, with Dunnett’s T3 multiple comparison test in Graphpad Prism v. 8.3.0.

### 4.3. Western Blot

Plasma samples from the challenge study were used in Western blot (WB) analyses. Samples with low Ct were chosen, with *n* = 2 from each of 2, 4, 6 and 8 wpc, and 14 µL plasma diluted 1:25 in PBS mixed with XT buffer and XT reducing agent (Bio-Rad, Hercules, CA, USA )was heated for 5 min at 95 °C and then loaded onto a 4–12% criterion XT bis-tris gel. Separated proteins were transferred onto a polyvinylidene difluoride (PVDF) membrane with Transblot Turbo (Bio-Rad) at 15 V for 30 min. The membranes were blocked with 3% bovine serum albumin (BSA) and 0.1% Tween 20 in PBS, and incubated overnight at 4 °C with antiserum against PRV-1 σ1 (1:500) [12], σ3 (1:500) [40], and µNS (1:1000) [24]. Horseradish peroxidase (HRP)-conjugated anti-rabbit IgG (Amersham, Buchinghamshire, UK) (1:20,000) was used as a secondary antibody. The Clarity Western ECL Substrate kit was used for immunodetection (Bio-Rad), and Precision Plus Protein as the molecular weight ladder (Bio-Rad). Images were acquired using ChemiDoc XRS+ system and Image One software (Bio-Rad). 

### 4.4. Flow Cytometry

Blood cells from the heparinized blood samples (*n* = 6) were analyzed for PRV-1 σ1 protein by flow cytometry, as previously described [2]. In brief, 50 μL of heparinized blood was diluted 1:20 in staining buffer and transferred to 96-well plates. The cells were fixed in intracellular (IC) fixation buffer (eBioscience, San Diago, CA, USA) and washed in permeabilization buffer. The cells were stained with rabbit polyclonal PRV-1 σ1 antibody (1:5000) for 30 min [12], washed, and then incubated with anti-rabbit IgG Alexa Fluor 488 secondary antibody (Molecular Probes, Eugene, OR, USA) (2 mg/mL diluted 1:800) for 30 min. The cells were counted in Gallios Flow Cytometer (Beckman Coulter, Miami, FL, USA), and the data analyzed using Kaluza software (Becton Dickinson).

### 4.5. Histology and Immunohistochemistry

Formalin-fixed, paraffin-embedded (FFPE) heart tissues (*n* = 6) were stained with hematoxylin and eosin. The blocks were blinded, and the histopathological changes in the heart sections were scored using a visual analog scale, as previously described [2]. Immunohistochemical staining of PRV-1 σ1 protein was standardized with RNAscope reagents. For immunohistochemistry, 4 µm sections were mounted on microscopic glass slides (Superfrost Plus, Thermo Fisher Scientific, Waltham, MA, USA) and deparaffinized for 60 min at 60 °C, followed by two changes in xylene and absolute ethanol for 5 min each. The sections were incubated with 3% hydrogen peroxide for 10 min to inactivate endogenous peroxidase, and then washed in distilled water. Antigen retrieval was performed at 95–99 °C for 15 min in RNAscope target retrieval reagent. Slides were then allowed to dry at room temperature, and a hydrophobic barrier was created around the sections using an ImmEdge Hydrophobic Barrier Pen (Vector Labs, USA). Sections were blocked with normal goat serum diluted 1:50 in PBS containing 5% BSA. Positive and negative control samples from previous challenge studies and non-infected samples (0 wpc), respectively, were also included. Slides were incubated overnight at 4 °C with PRV-1 σ1 antiserum [12], diluted in PBS (1:3000) containing 1% BSA. The slides were then washed with PBS and incubated with HRP-labelled goat anti-rabbit secondary antibody from the Dako EnVision kit. In the final step, slides were incubated with the diaminobenzidine tetrahydrochloride (DAB) substrate chromogen for 5 min for color reaction (brown) and counterstained with hematoxylin. 

### 4.6. In Situ Hybridization 

RNAscope in situ hybridization (ISH) protocols targeting the PRV-1 segment L3 region 415-1379 (relative to ORF start) (KY429945) (catalogue number 537451) and the predicted *Salmo salar* arginase-2 gene (ARG2) (XM_014190234) region 1332-2053 were developed using the RNAscope 2.5 HD detection red kit (Advanced Cell Diagnostics, Newark, CA, USA), as previously described [16]. A 20 ZZ probe pair targeting *Salmo salar* peptidylprolyl isomerase B (ppib) (PPIB) mRNA (catalogue number 494421) was used as a control of RNA quality, and a probe against the *Bacillus subtilis* strain SMY methylglyoxal synthase (mgsA) gene (catalogue number 310043) was used as negative control. 

In brief, FFPE sections were deparaffinized at 60 °C for 90 min, and then transferred to xylene and twice to absolute ethanol, incubating 5 min each time. The inactivation of endogenous peroxidase was performed with 3% hydrogen peroxide for 10 min at room temperature. The sections were then boiled for 15 min in RNAscope target retrieval buffer, followed by a 15 min incubation at 40 °C with RNAscope protease reagent. Hybridization was performed with PRV-1 probe for 2 hrs at 40 °C. Slides were incubated with amplifiers (amp 1 to amp 6), with a duration time recommended by the manufacturer, and the signals were detected with Fast red. Slides were counterstained with 50% Gill’s Hematoxylin I for 10 min and mounted in EcoMount. For dual staining of ISH and IHC, after antigen retrieval, slides were incubated with protease (RNAscope Protease Plus) for 10 min and stained for ISH, followed by blocking and then staining for IHC.

## Supplementary Materials

The following are available online at https://www.mdpi.com/2076-0817/9/2/143/s1, Figure S1. ISH-positive control sections stained with Atlantic salmon (*Salmo salar*) peptidylprolyl isomerase B mRNA. Negative control sections are stained with a probe against the Bacillus subtilis strain SMY methylglyoxal synthase (mgsA) gene. Figure S2. Immunohistochemistry (IHC) positive control for Atlantic salmon (*Salmo salar*) heart sections and negative control tissues stained with PRV-1 σ1 antibody.
