# Supplementary material for: Dissemination of Piscine orthoreovirus-1 (PRV-1) in Atlantic Salmon (Salmo salar) during the Early and Regenerating Phases of Infection
Source: Pathogens. 2020 Feb 20;9(2):143. doi: 10.3390/pathogens9020143 (PMC7169402; doi:10.3390/pathogens9020143)
Supplement: Supplementary file 1 [file pathogens-09-00143-s001.pdf]

Supplementary figures

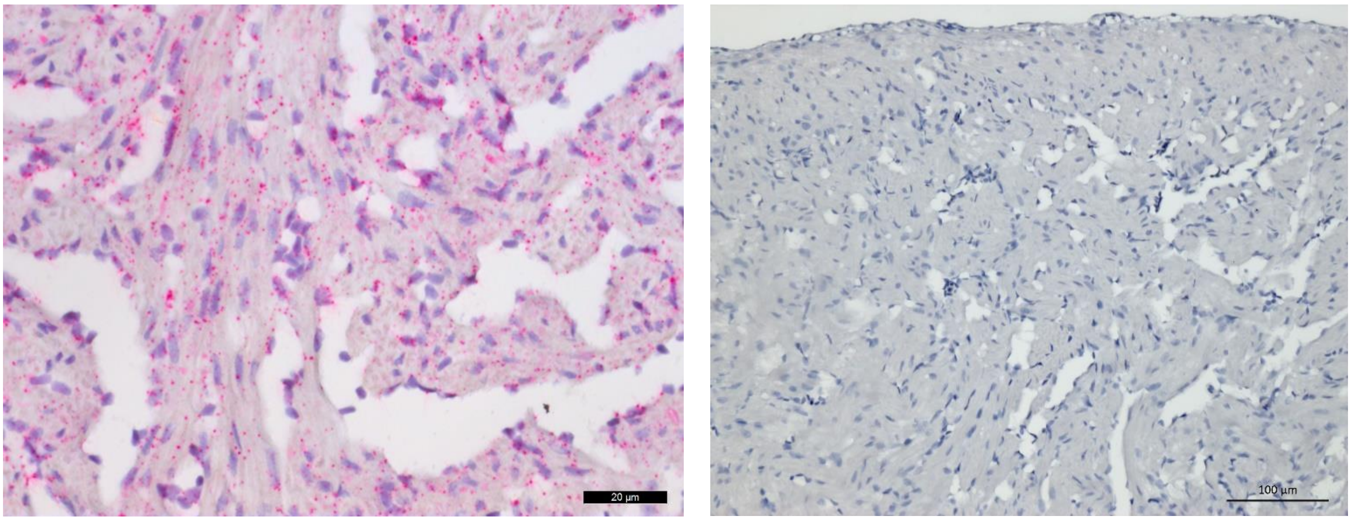

Figure S1. ISH positive control sections stained with Atlantic salmon (*Salmo salar*) peptidylprolyl isomerase B mRNA. Negative control sections stained with a probe against the *Bacillus subtilis* strain SMY methylglyoxal synthase (mgsA) gene.

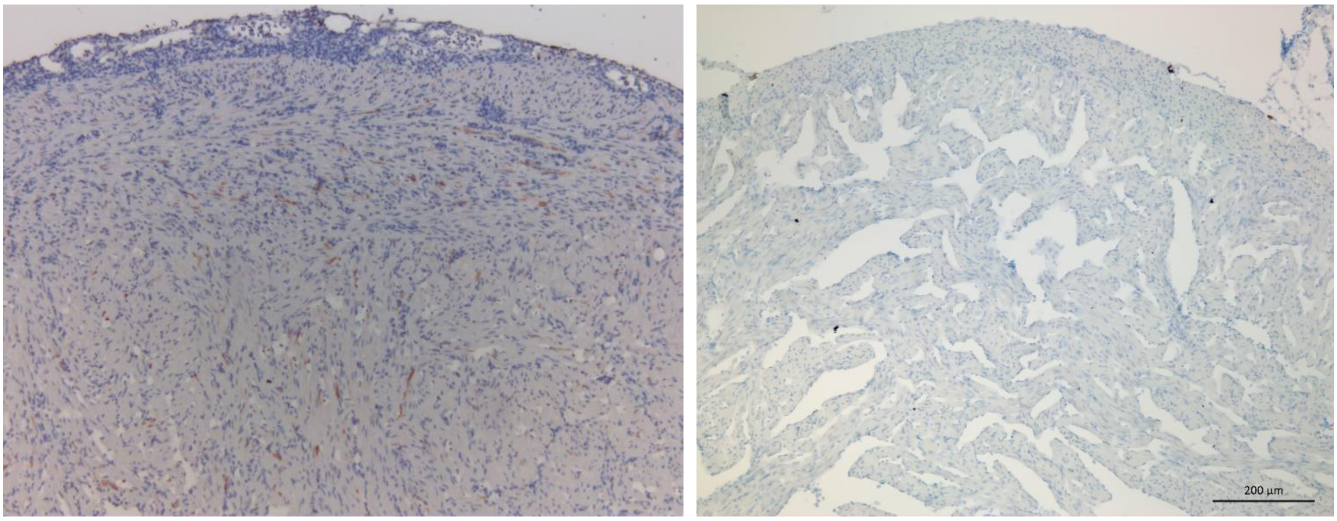

Figure S2. Immunohistochemistry (IHC) positive control Atlantic salmon (*Salmo salar*) heart sections and negative control tissues stained with PRV-1 σ1 antibody.
